# Emerging Pollutants in the Environment: Occurrence, Fate, Risk Assessment and Degradation Methods

**DOI:** 10.3390/toxics13070521

**Published:** 2025-06-22

**Authors:** Qingwei Bu, Yuning Ma

**Affiliations:** 1 School of Chemical & Environmental Engineering, China University of Mining & Technology-Beijing, Beijing 100083, China; 2 College of Environmental and Resource Sciences, Zhejiang University, Hangzhou 310058, China; julius.yuningma@gmail.com

## 1. Introduction

The use of chemical substances has brought great benefits to human well-being; however, the dark side of the coin is that they may pose risks to human health and the environment. As a consequence of the uncontrolled or unregulated use of many anthropogenic chemicals, humanity has exceeded the safe operating space (defined as the planetary boundary) for these chemicals [1,2]. In this context, emerging pollutants (also commonly known as emerging contaminants) are defined as chemicals that are not currently regulated but raise ecological or human health concerns. These pollutants include, but are not limited to, endocrine-disrupting compounds, pharmaceutical and personal care products, disinfection by-products, microplastics, and persistent organic chemicals, etc.—along with their degradation products—detected in air, water, soil, and food sources [3].

In recent decades, extensive efforts have been exserted to understand the occurrence, fate, and toxicity of these chemicals, as well as to develop new technologies for mitigating the associated risks. This Special Issue presents the most recent advancements in emerging pollutant research, including studies on their occurrence, distribution, fate, and the associated environmental risks, along with technological developments aimed at reducing and controlling their environmental presence.

## 2. An Overview of Published Articles

There are seven articles focused the occurrence and associated ecological/health risks of typical emerging pollutants, e.g., antibiotics, pesticides, and endocrine-disrupting chemicals.

Industrial activities heavily rely on organic solvents, which release volatile organic compounds (VOCs) linked to environmental pollution and occupational health hazards, particularly in manufacturing sectors like electronics and chemicals. Guo et al. (contribution 2) investigated the temporal trends and industry-specific differences in VOC usage and the associated health risks in Bao’an District, Shenzhen, China, from 2018 to 2023. Analyzing 1335 solvent samples and 1554 air samples, the research found that VOC usage declined during the COVID-19 pandemic but rebounded thereafter. Alkanes and aromatic hydrocarbons (e.g., toluene, n-hexane, and xylene) were the most prevalent VOCs, with detection rates highlighting toluene (22.5%) and n-hexane (22.0%) as the dominant components. Air monitoring identified trichloroethylene and xylene as high-risk compounds, exceeding acceptable health thresholds. Post-2020 data revealed a trend toward reduced solvent diversity, potentially lowering mixed-exposure risks but raising concerns about new substitutes. The study underscores the need for the targeted monitoring of high-risk VOCs and improved workplace safety measures to mitigate occupational hazards in industrial regions.

E-waste dismantling is a significant source of bisphenol chemicals (BPs) due to the release of additives from plastics and epoxy resins during processing. Zhao et al. (contribution 3) investigated the spatial distribution and health risks of BPs in surface soil from e-waste dismantling facilities and surrounding areas in South China. Using non-targeted screening and targeted analysis, 14 BPs were identified, including bisphenol A (BPA), tetrabromobisphenol A (TBBPA), and novel structural analogs. Total BP concentrations in e-waste soil (median: 6970 ng/g) far exceeded those in surrounding areas (median: 197 ng/g), with BPA, TBBPA, and bisphenol F being dominant. Spatial analysis revealed declining TBBPA and its debromination product concentrations with increasing distance from e-waste sites, indicating facility emissions as a primary source. Risk assessment via soil ingestion showed daily intakes for workers and residents were below current tolerable thresholds; the exception being BPA in workers, which surpassed stricter recent guidelines. The findings highlight e-waste activities as critical BP emission sources, necessitating ongoing monitoring to address potential health risks from emerging analogs and cumulative exposures. In another study, by Chen et al. (contribution 6), the authors investigated the contamination of BPs in aquatic products from South China, focusing on human exposure risks. The researchers analyzed 245 samples (of fish, crustaceans, and bivalves) from Shenzhen markets using liquid chromatography–mass spectrometry. All BPs except bisphenol AF were detected, with bisphenol S showing the highest detection rate. Enzymatic hydrolysis revealed that 49–96% of the BPs existed in bound forms, significantly increasing post-treatment concentrations. Bisphenol F and bisphenol S dominated the contamination profiles, likely due to their being increasingly used as BPA substitutes. Correlation analysis suggested shared pollution sources for certain BPs, and health risk assessments indicated low exposure risks, though females exhibited slightly higher risks than males. The study underscores the necessity of enzymatic hydrolysis for accurate BP detection and highlights the need for stricter regulations to mitigate contamination, despite the health threat currently being low.

Yang et al. (contribution 4) addressed the urgent need to regulate emerging pollutants in wastewater treatment plant effluents in China, focusing on pharmaceuticals and endocrine-disrupting chemicals. These pollutants, detected in aquatic ecosystems globally, pose ecological and human health risks due to their inadequate removal by conventional treatment processes. Analyzing data from 2012 to 2022, the research team identified 140 emerging pollutants in Chinese wastewater treatment plant effluents, with concentrations ranging from undetected levels to 706 μg/L. High-risk regions included Gansu, Hebei, Shandong, Guangdong, and Hong Kong. Using risk assessment methods, eighteen high-risk emerging pollutants were prioritized, but only carbamazepine, ibuprofen, and BPA met the conditions needed to derive long-term water quality criteria via species sensitivity distribution—their long-term water quality criteria values were 96.4, 1010, and 288 ng/L, respectively. Notably, carbamazepine and BPA concentrations frequently exceeded these thresholds, highlighting critical regulatory gaps. The study underscores the necessity for targeted monitoring and science-based discharge limits to mitigate ecological and health impacts, providing a foundational framework for the management of emerging pollutants in China’s urban wastewater systems.

Dimethylcyclosiloxanes, widely used in silicone polymers for their thermal stability and flexibility, are linked to endocrine disruption, reproductive toxicity, and environmental persistence. Xing et al. (contribution 5) investigated the environmental and health implications of dimethylcyclosiloxanes in mobile smart terminal devices, focusing on their concentrations, distribution patterns, and emissions. Analyzing silicone rubber, adhesives, and plastics from devices like headphones and smartphones, the researchers found D5–D9 to be prevalent in silicone rubber (detection rates: 91–95.5%) and adhesives (50–100%), with total concentrations reaching 802.2 mg/kg in silicone rubber. Meanwhile, plastics exhibited higher detection rates of the lower-weight D3 and D4 (61.1%). Environmental emissions from silicone rubber in China were estimated to exceed 5000 tons annually, raising concerns about long-term ecological and human exposure. The study highlights the dominance of the understudied higher-weight D7–D9 in silicone materials and calls for toxicological assessments of these compounds. It underscores the need for optimized manufacturing processes to reduce residual dimethylcyclosiloxanes and improved regulatory frameworks to mitigate risks from widespread device disposal and environmental release.

With extensive agricultural activity around Xingkai Lake—a critical freshwater ecosystem—pesticide residues pose potential threats to water quality and aquatic life. Wang et al. (contribution 9) investigated the distribution and ecological risks of 57 pesticides in farmland soil and surrounding water bodies in the Xingkai Lake area, Heilongjiang Province, China. The researchers analyzed soil and water samples across three periods (sowing, vegetative, and maturity stages). Their key findings revealed 43 pesticides and 3 degradation products in the soil, with atrazine and acetochlor dominant in dry fields, while oxadiazon, mefenacet, and chlorpyrifos prevailed in paddy fields. The analyzed water samples showed peak contamination during the vegetative period, with atrazine, simetryn, and buprofezin as primary pollutants in drainage and lake water. Correlation analysis (*r* > 0.8) indicated shared contamination sources between the drainage systems and the lake, and ecological risk assessments highlighted significant risks from atrazine, chlorpyrifos, and prometryn, with potential affected species fractions exceeding 5%. The study underscores the impact of agricultural pesticides on freshwater ecosystems, emphasizing the need for targeted management to mitigate long-term ecological harm in ecologically sensitive regions like Xingkai Lake.

Organophosphorus flame retardants (OPFRs) are widely detected in indoor dust and air due to their additive nature in consumer products like furniture and electronics, leading to prolonged human exposure through ingestion, inhalation, and dermal contact. Song et al. (contribution 10) reviewed the distribution and health impacts of OPFRs in indoor environments, focusing on their role as replacements for restricted polybrominated diphenyl ethers. Key findings highlight higher concentrations of OPFRs in dust compared to air, with tris(2-butoxyethyl) phosphate, tris(1-chloro-2-propyl) phosphate, and tris(1,3-dichloro-2-propyl) phosphate being predominant. Regional variations in OPFR profiles reflect differences in usage patterns and regulations, such as increased tris(1-chloro-2-propyl) phosphate levels in Europe following tris(2-chloroethyl) phosphate restrictions, and health risk assessments indicate toddler exposure via dust ingestion in Japan reaching concerning levels, though overall combined exposure typically remains below reference thresholds. The study underscores the need for integrated evaluations of OPFR toxicity and exposure pathways, proposing pollutant equivalency factors to prioritize risk management, and provides a foundational framework for understanding OPFR-related health risks and informing regulatory strategies.

The remaining three articles focused on developing treatment technologies for the removal of emerging pollutants.

Silva et al. (contribution 1) addressed the environmental challenge posed by tenofovir disoproxil fumarate (TDF), a widely used antiretroviral drug for HIV treatment, whose stable metabolite, tenofovir, persists in aquatic ecosystems and poses risks to aquatic organisms. The authors evaluated the biodegradation potential of a cyanobacteria–bacterial consortium (*Microcystis novacekii* and *Pseudomonas pseudoalcaligenes*) for TDF removal. The process occurred in two phases—abiotic and enzymatic de-esterification of TDF into tenofovir monoester (TMF) within 72 h, followed by the intracellular removal of TMF over 16 days. The consortium achieved a 88.7–94.1% removal efficiency across TDF concentrations (12.5–50 mg/L), with optimal performance at 25 mg/L. Notably, tenofovir itself was not detected, but residual TMF—a partially active antiviral intermediate—remained, highlighting incomplete degradation. The findings underscore the potential of microbial consortia for sustainable pharmaceutical wastewater treatment while emphasizing the need for further research to address persistent metabolites. This work contributes to strategies for mitigating pharmaceutical pollution in aquatic environments, particularly for high-persistence drugs like TDF.

Conventional methods like advanced oxidation processes are energy-intensive and costly, prompting the exploration of adsorption using carbon aerogels (CAs) as a sustainable alternative. Lu et al. (contribution 7) addressed the environmental challenge of removing 1,4-dioxane, a carcinogenic and highly water-miscible pollutant, from contaminated water. The research synthesized CAs through controlled pyrolysis conditions (e.g., temperature and heating rate) to optimize their porous structure, achieving a specific surface area of 673.89 m^2^/g with enhanced mesoporosity. The optimized CAs demonstrated exceptional adsorption performance, removing over 95% of 1,4-dioxane, following quasi-second-order kinetics and Langmuir isotherm models, indicating monolayer adsorption. The maximum capacity reached 67.28 mg/g at 318 K, which was attributed to the material’s mesoporous network and microporous synergy. Notably, competitive adsorption tests with trichloroethylene showed no significant inhibition, and regeneration experiments confirmed stable performance over five cycles. These findings highlight CAs as a cost-effective, reusable adsorbent for water purification, offering a practical solution for persistent organic pollutants like 1,4-dioxane while minimizing secondary environmental impacts.

Traditional methods like membranes and adsorbents face limitations in flexibility and degradability. Yu et al. (contribution 8) addressed the challenge of VOC emissions during soil remediation, which pose significant health and environmental risks. The research introduces an aqueous foam stabilized by SiO_2_-TiO_2_ nanoparticles to enhance VOC suppression. By modifying silica nanoparticles with hydrophobic groups and integrating TiO_2_ for photocatalysis, the foam exhibits improved stability and functionality, and the experimental results show that the modified nanoparticles increase foam liquid half-life by 4.08 h and volume half-life by 4.44 h, compared to nanoparticle-free foam. Under UV irradiation, the foam maintained a 90% suppression rate for dichloroethane, n-hexane, and toluene for nearly 12 h, outperforming other variants. Characterization confirmed enhanced dispersibility and oxygen vacancies in the composite nanoparticles, boosting photocatalytic efficiency. This innovation offers a dual-function solution—blocking VOC emissions while degrading contaminants—providing an eco-friendly, adaptable alternative for soil remediation with reduced secondary pollution risks.

## 3. Future Development Prospects

The collection of articles in this Special Issue provides valuable contributions to our understanding of the occurrence and risk profiles of emerging pollutants; however, several critical research gaps warrant attention. Notably, there is a paucity of systematic investigations into ecotoxicological impacts, epidemiological correlations, exposomic interactions, and metabolic pathways, which are all crucial dimensions of comprehensive risk assessment. These aspects present significant challenges for environmental researchers given the diverse physicochemical properties and biological activities exhibited by the expanding spectrum of emerging pollutants. Particularly noteworthy are compounds with distinct mechanisms of action, such as endocrine disruptors, which require specialized evaluation frameworks. Furthermore, while three papers addressed contaminant mitigation technologies, there remains a notable absence of research on advanced oxidation processes in this collection. This omission is particularly conspicuous as advanced oxidation processes represent state-of-the-art advancements in contaminant degradation and are poised to remain a focal point of environmental engineering research given their effectiveness in addressing persistent organic pollutants.

## Data Availability

Not applicable.
